# Rebalancing meat and legume consumption: change-inducing food choice motives and associated individual characteristics in non-vegetarian adults

**DOI:** 10.1186/s12966-022-01317-w

**Published:** 2022-09-01

**Authors:** Anouk Reuzé, Caroline Méjean, Myriam Carrère, Lucie Sirieix, Nathalie Druesne-Pecollo, Sandrine Péneau, Mathilde Touvier, Serge Hercberg, Emmanuelle Kesse-Guyot, Benjamin Allès

**Affiliations:** 1 Université Paris Cité, CRESS, INSERM, INRAE, CNAM, Bobigny, F-93017 France; 2 Équipe de Recherche en Épidémiologie Nutritionnelle (EREN) - Centre de Recherche en Épidémiologie et StatistiqueS (CRESS), Université Sorbonne Paris Nord, Inserm U1153, Inrae U1125, Cnam FR SMBH, 74, rue Marcel Cachin, F-93017 Bobigny, France; 3 MOISA, Université de Montpellier, CIRAD, CIHEAM-IAMM, INRAE, IRD, Institut Agro, Montpellier, France; 4 Department of Public Health, AP-HP Hôpital Avicenne, 93017 Bobigny, France

**Keywords:** Sustainability, Food motives, Food behavior change, Epidemiology, Social marketing

## Abstract

**Background:**

A shift toward more plant-based foods in diets is required to improve health and to reduce environmental impact. Little is known about food choice motives and associated characteristics of those individuals who have actually reduced their consumption of animal-based foods. The aim of this cross-sectional study was to identify change-inducing motives related to meat and legume consumptions among non-vegetarians. The association between change-inducing motives and individual characteristics was also studied.

**Methods:**

This study included 25,393 non-vegetarian participants in the French NutriNet-Santé cohort (77.4% women, mean age 55.4 ± 13.9 y.). The motives related to the declared change in meat and legume consumptions (e.g., taste, environment, social pressure) were assessed by an online questionnaire in 2018. For each motive, respondents could be classified into three groups: no motive; motive, not change-inducing; change-inducing motive. Associations between change-inducing motives and individual characteristics were evaluated using multivariable polytomic logistic regressions. Characteristics of participants who rebalanced their meat and legume consumptions were also compared to those who reduced their meat but did not increase their legume consumption.

**Results:**

Motives most strongly declared as having induced a change in meat or legume consumptions were health and nutrition (respectively 90.7 and 81.0% declared these motives as change-inducing for the meat reduction), physical environment (82.0% for meat reduction only) and taste preferences (77.7% for legume increase only). Other motives related to social influences, meat avoidance and meat dislike were reported by fewer individuals, but were declared as having induced changes in food consumption. Most motives that induced a meat reduction and a legume increase were more likely to be associated with specific individual characteristics, for example being a woman or highly educated for health motives.

**Conclusions:**

Besides the motives reported as important, some motives less frequently felt important were declared as having induced changes in meat or legume consumptions. Change-inducing motives were reported by specific subpopulations. Public campaigns on health and sustainability could usefully develop new tools to reach populations less willing to change.

**Trial registrations:**

The study was registered at ClinicalTrials.gov (NCT03335644).

**Supplementary Information:**

The online version contains supplementary material available at 10.1186/s12966-022-01317-w.

## Background

A shift toward a smaller contribution of animal-based foods to human diets is required to improve health and reduce the environmental impact of diet [1]. One such dietary transition pathway is a rebalance of animal and plant food consumption, namely reducing meat and increasing plant foods, such as legumes, cereals, fruits and vegetables. However, very few studies have been conducted in non-vegetarians on the potential transition to a diet including more plant-based foods.

Among plant-based foods, legumes have been recognized as a sustainable source of dietary protein [2]. However, not all plant-based foods may be socially desirable, as is currently the case for legumes in many developed countries [3]. For instance, in Europe, the average consumption of legumes is estimated at 7 g per capita per day, which is very low (estimated in the early 2010s [3]). Reducing meat and increasing legumes thus remains a challenge for a sustainable nutrition transition.

To efficiently promote a dietary transition, a better understanding of what influences the changes in food consumption is required. Some previous studies have highlighted food choice motives – defined as “factors thought to influence people’s dietary choices” [4] (abbreviated to “motives”) – related to health, taste preferences, environment, animal protection, or price that were frequently reported for reduced meat consumption [5–9] and increased plant-based food consumption [10]. Other food choice motives related to practical issues such as food convenience and accessibility, and social influences such as habits, social pressures, social norms, and social representations also seem to influence meat consumption [11]. In addition to studies exploring food choice motives, some theories have been used to investigate determinants of changes in animal-based and plant-based food consumptions [6, 8, 10, 12–14]. For example, using the theory of planned behavior, attitudes toward meat consumption (i.e., “evaluation of the pros and cons of performing the behaviour” [8]) are good predictors of intention to reduce meat consumption [8, 12, 13]. However, most of these theories predict what factors influence the behavior depending on assumptions and aims [15], which is not the case for food choice motives.

**Fig. 1:**
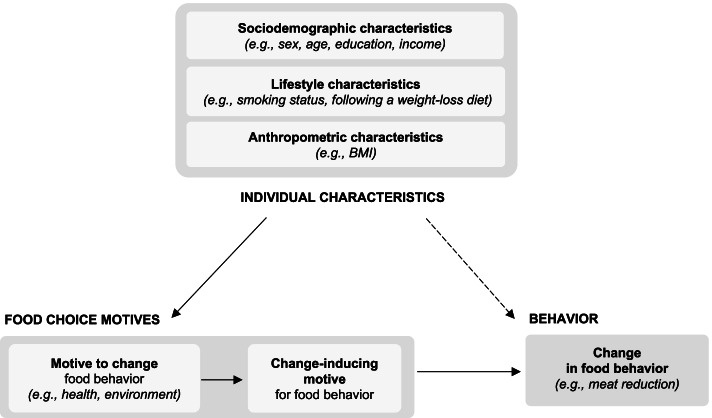
Conceptual scheme of food choice motives inducing a change in food consumption Individual sociodemographic, anthropometric, and lifestyle characteristics were associated with change in food behavior. These characteristics were also associated with motives that led to a change in behavior. However, we assumed that only change-inducing motives would induce this change in food consumptions. Dotted lines correspond to what had already been investigated in previous studies on the sociodemographic determinants of food behavior, such as those related to the consumption of animal products [16]

Although many potential motives have been identified by previous studies, more information is needed on what actually induce a change in behavior. For example, preservation of the physical environment could be a concern, for individuals who are reducing their meat consumption, yet these individuals may not be changing their behavior for that reason. We hypothesized that among all the many motives, some were declared more effective in inducing a change (change-inducing motives) in meat and legume consumption than others (see Fig. 1), and could form subgroups with specific characteristics.

The aim of this cross-sectional study was to describe change-inducing motives related to the consumption of meat and legumes, in a population of non-vegetarian French adults from the NutriNet-Santé cohort. A further aim of the study was to describe the association between the change-inducing motives and sociodemographic and lifestyle characteristics.

## Methods

### Study population

The NutriNet-Santé study is a web-based prospective observational French cohort launched in May 2009. It investigates the relationship between nutrition and health, especially chronic disease risk, and the determinants of dietary behavior and nutritional status. Briefly, participants are Internet-using adult volunteers prospectively recruited among the general population. The study design has been described elsewhere [17]. It was conducted according to the guidelines laid down in the Declaration of Helsinki and was approved by the Institutional Review Board of the French Institute for Health and Medical Research and the Commission Nationale de l’Informatique et des Libertés (CNIL 908,450 and 909,216). All the participants signed an electronic informed consent statement. The Clinical Trial number is NCT03335644.

### Change-inducing motives related to meat and legume consumptions

A questionnaire addressing the motives for changing consumption of animal-based and plant-based foods in diet was developed, based on a previous questionnaire on food choice motives [18–20] and with a multidisciplinary collaboration of epidemiology, nutrition, and social marketing researchers – defined as the science that seeks to develop and integrate marketing concepts with other approaches to influence behaviors that benefit individuals and communities for the greater social good [21]). This supplemental questionnaire was sent in August 2018 to NutriNet-Santé participants. Participants were asked to declare whether they were following a vegetarian or vegan diet, with those answering the affirmative being excluded from the study (final participation rate of 24.6%, based on the whole NutriNet-Santé study since its launch). As meat, particularly ruminant meat, is a major concern for food sustainability and human health [1], we focused the attention of participants on it, defining the term “meat” as beef, minced or roast steak, rib steak, stew, ground beef in a dish such as lasagna or spaghetti Bolognese, veal, lamb, pork, offal, breaded meat, game, rabbit or hare and all processed meats or derived products. This definition excluded poultry (turkey, chicken, duck, quail, pigeon). Legumes were defined as the food group including fava beans, flageolets, white, black and red beans, lentils, lupins, split peas, chickpeas, soy protein, tofu and tempeh.

#### Changes in consumption

The participants were asked to respond to the following statements by “yes” or “no”: “*I have reduced, or already thought about reducing my meat consumption*”, “*I have always maintained my meat consumption, I have never felt like reducing it*”, “*I have reduced or thought about reducing my legume consumption, or, at least, I am not trying to increase it*”, “*I have increased or already thought about increasing my legume consumption*”. If participants gave a favorable response to one statement, we labelled it as a declared change in consumption. Based on responses to previous statements, we also differentiated participants who declared that they had reduced or already thought about reducing their consumption of meat into two groups, according to how they stood with regard to an increase in legumes: (i) those who had also increased or already thought about increasing their consumption of legumes, as a rebalancing of their consumption of meat and legumes, (ii) those who did not declare any increase in their legume consumption.

#### Change-inducing motives

For each type of change in consumption, a set of 12 motives were proposed, including taste, health, environment, animal protection, and sociocultural influences (see all items in Additional file 1). We used existing literature for the selection of motives in the questionnaire [6, 22]. Free text responses to indicate additional motives were not considered in this list.

##### Motives

For each motive, participants who declared a change in their meat/legume consumption were asked to rate their corresponding motives on a 5-point Likert scale, from “*Strongly disagree*” to “*Strongly agree*”, including “*Neither agree nor disagree*”, plus an “*I don’t know*” answer. For example, participants who declared a reduction of their meat consumption, were asked to rate statements such as “*I care about animal welfare or the lives of animals*” or “*I think it’s healthier not to eat too much meat*”.

##### Change-inducing motives

If participants gave a favorable response to one motive (“*Somewhat agree*” or “*Strongly agree*”), a second statement “*and it encourages me to reduce/increase my meat/legume consumption*” was proposed to assess if this motive induced a change in consumption, on a separate 5-point Likert scale.

Both Likert scales, for the motive and for the change-inducing motive, were then recoded to compute agreement scores ranging from 0 to 5 and 1 to 5, respectively.

##### Groups of motives

Three groups were obtained based on the two questions on motives:

- “No motive”: Participants were considered to have “no motive” if they gave an unfavorable response (“*Strongly disagree*”, “*Somewhat disagree*”, “*Neither agree nor disagree*” and “*I don’t know*”) for the motive. For this group, the given motive was thus not felt important.
- “Motive, not change-inducing” : Participants with a “motive, not change-inducing” were those who gave a favorable response (“*Strongly agree*” and “*Somewhat agree*”) to the motive but an unfavorable one (“*Strongly disagree*”, “*Somewhat disagree*”, “*Neither agree nor disagree*”) to the statement that the motive had induced a change in consumption. For this group, the given motive was thus felt important but was not declared as change-inducing.
- “Change-inducing motive”: Participants who gave favorable responses to both sets of statements were considered as having a “change-inducing motive”. For this group, the given motive was thus felt important and did lead to a change.

### Sociodemographic, anthropometric, and lifestyle data

At baseline and once a year thereafter, participants were invited to fill out a set of self-administered questionnaires on sociodemographic, anthropometric, and lifestyle characteristics. Data collected included sex, age, socioprofessional category (unemployed/self-employed, farmer, employee, manual worker/intermediate profession/managerial staff, intellectual profession/no occupation), educational level (none or primary/secondary/undergraduate and others/postgraduate), household composition (alone without children/alone with at least one child/two adults living as a couple without children/ two adults living as a couple with at least one child/two or more adults without children), size of the urban residence unit (rural/< 20,000 inhabitants/20,000–200,000 inhabitants/> 200,000 inhabitants). Monthly income per household unit was obtained per household consumer unit (CU). One CU is assigned to the first adult in the household, 0.5 CU for other persons aged 14 or older and 0.3 CU for children under 14. Five categories were defined and were assigned to participants: < 1200 € per c.u./1200–1800 € per c.u./1800–2700 € per c.u./> 2700 € per c.u./Refused to declare). The date of the latest weight-loss diet followed was collected, and individuals were classified into three groups: no declared diet, < 5 years, > 5 years. Self-reported height and weight measurements were validated against clinical measurements [23]. Body mass index (BMI) was calculated as weight (kg) per height squared (m^2^), and participants were divided according to World Health Organization (WHO) criteria: underweight (< 18.5 kg/m^2^), normal (18.5–25 kg/m^2^), overweight (excluding obesity) (25–30 kg/m^2^), obese (≥30 kg/m^2^) [24].

### Statistical analyses

**Fig. 2:**
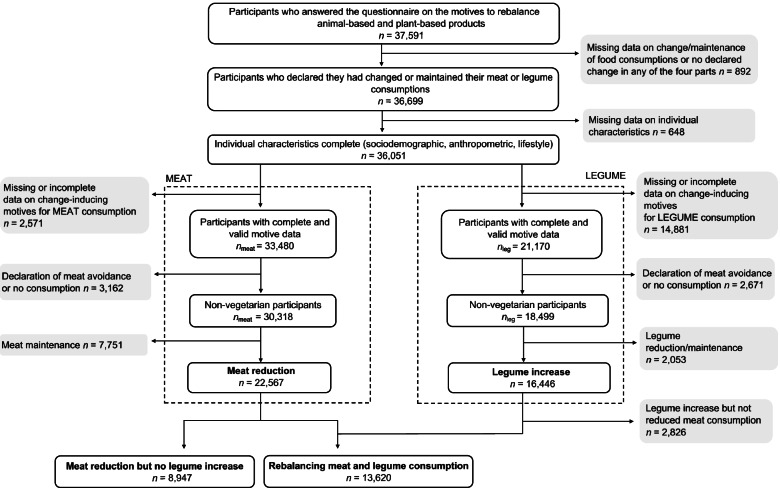
Flowchart of the study

We included participants who completed at least one section of the supplemental questionnaire on meat reduction and legume increase, and who gave complete sociodemographic, anthropometric, and lifestyle data. Participants who self-declared as vegans or vegetarians, and those who completed only the section on the maintenance of meat consumption or only on the reduction/maintenance of legume consumption were excluded. The flow chart is presented in Fig. 2.

Sociodemographic and lifestyle characteristics were compared between the samples of included and excluded participants, and were described for the samples of meat reduction and legume increase. The characteristics of participants who had rebalanced their consumption of meat and legumes were compared to those who declared a meat reduction but did not declare an increase in legumes, using logistic regression models. Participants who only declared a legume increase were not considered in this analysis.

For each motive, when it was felt important, we calculated the frequency of participants who declared this motive as change-inducing.

For each item, multivariable logistic regression models were applied to assess the association between groups of motives and individual characteristics (with the “no motive” group as reference). These models were adjusted for all characteristics taken together (sex, age, household income, socioprofessional category, educational level, household composition, BMI, size of the urban residence unit, and latest weight-loss diet followed).

All tests were two-sided, and a *p*-value < 0.05 was considered significant. Statistical analyses were conducted with SAS (version 9.4, SAS Institute, Inc.).

## Results

### Sample selection and description

A total of 25,393 participants of NutriNet-Santé were included in the present analysis. The total sample included individual who both declared a meat reduction and legume increase (*n* = 13,620, 53.6%), those who only declared a meat reduction (*n* = 8947, 35.3%) and those who only declared a legume increase (*n* = 2826, 11.1%). Compared to participants who declared a meat reduction (*n* = 22,567), participants who declared an increase in legumes (*n* = 16,446) included more women, younger participants, more participants in a higher socioprofessional category, with a higher educational level, living as a couple with children and in bigger cities, and more participants who did not declare a weight-loss diet (Table 1).

**Table 1:** Sociodemographic and lifestyle characteristics of included and excluded samples, and meat reduction and legume increase samples, NutriNet-Santé 2009–2018, *n* = 37,591

	Included (***n*** = 25,393)		Excluded (***n*** = 12,198)			Meat reduction (***n*** = 22,567)		Legume increase (***n*** = 16,446)	
	***n***	%	***n***	%	*p*^1^	***n***	%	***n***	%
**Sex**					< 0.0001				
Men	5741	22.6	3523	28.9		5138	22.8	3345	20.3
Women	19,652	77.4	8675	71.1		17,429	77.2	13,101	79.7
**Age**					< 0.0001				
[18–30[	920	3.6	709	5.8		825	3.7	625	3.8
[30–50[	7522	29.6	3714	30.5		6711	29.7	5097	31.0
[50–65[	8934	35.2	3830	31.4		8014	35.5	5841	35.5
[65 + [	8017	31.6	3945	32.3		7017	31.1	4883	29.7
**Monthly household income classes**					< 0.0001				
< 1200 €	3444	13.6	2093	17.2		3036	13.5	2198	13.4
1200–1800 €	5029	19.8	2684	22.0		4445	19.7	3247	19.7
1800–2700 €	5884	23.2	2770	22.7		5251	23.3	3789	23.0
> 2700 €	8530	33.6	3133	25.7		7642	33.9	5624	34.2
Refused to declare	2506	9.9	1518	12.4		2193	9.7	1588	9.7
**Socioprofessional category**^2^					< 0.0001				
Self-employed, farmer, employee, manual worker	6497	25.6	3436	28.5		5686	25.2	3989	24.3
Intermediate profession	6201	24.4	2714	22.5		5478	24.3	4128	25.1
Managerial staff, intellectual profession	10,403	41.0	4124	34.2		9340	41.4	6831	41.5
No occupation	311	1.2	587	4.9		273	1.2	199	1.2
Unemployed	1981	7.8	1211	10.0		1790	7.9	1299	7.9
**Educational level**^2^					< 0.0001				
None or primary	406	1.6	299	2.5		344	1.5	214	1.3
Secondary	6410	25.2	3757	30.9		5635	25.0	3659	22.2
Undergraduate and others	8224	32.4	3713	30.6		7293	32.3	5491	33.4
Postgraduate	10,353	40.8	4376	36.0		9295	41.2	7082	43.1
**Household composition**^2^					< 0.0001				
Alone without children	4666	18.4	2507	20.8		4092	18.1	3024	18.4
Alone with at least one child	1712	6.7	882	7.3		1530	6.8	1073	6.5
Two adults living as a couple without children	10,526	41.5	4769	39.5		9351	41.4	6705	40.8
Two adults living as a couple with at least one child	8025	31.6	3444	28.5		7174	31.8	5344	32.5
Two or more adults without children	464	1.8	473	3.9		420	1.9	300	1.8
**Size of the urban residence unit**^2^					< 0.0001				
Rural	5467	21.5	2718	23.2		4856	21.5	3465	21.1
< 20,000 inhabitants	3846	15.2	1840	15.7		3411	15.1	2470	15.0
20,000–200,000 inhabitants	4678	18.4	2150	18.4		4156	18.4	2971	18.1
> 200,000 inhabitants	11,402	44.9	4987	42.6		10,144	45.0	7540	45.8
**Latest weight-loss diet followed**					< 0.0001				
No declared diet	9758	38.4	7717	63.3		8687	38.5	6441	39.2
< 5 years	1852	7.3	489	4.0		1656	7.3	1201	7.3
> 5 years	13,783	54.3	3992	32.7		12,224	54.2	8804	53.5

^1^*p* for chi^2^ test

^2^Among all the participants after exclusion of missing values

### Meat reduction

#### Change-inducing-motives for meat reduction

**Fig. 3:**
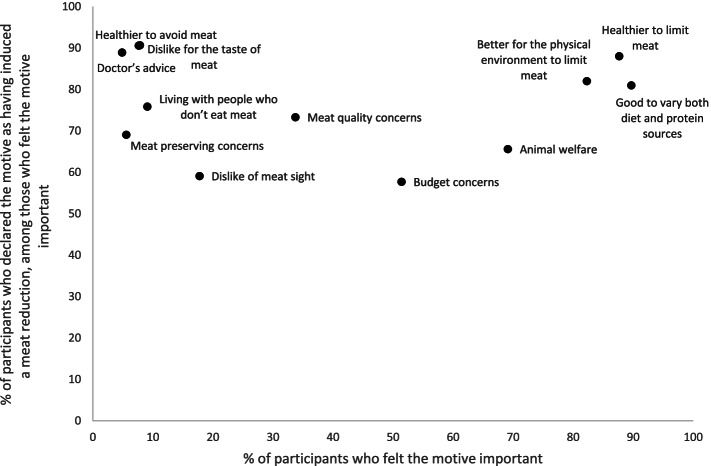
Comparison of the frequency of participants who declared the motive as having induced a meat reduction, according to the frequency of participants who declared the motive as important. Meat reduction sample. NutriNet-Santé study, 2018 (*n* = 22,567)

Among motives that were frequently felt important (> 82%), three were frequently declared as having induced a reduction of meat consumption (> 80%): “*good to vary both diet and protein sources”, “healthier”* and “*better for the physical environment to limit meat”* (Fig. 3). Of the motives less frequently felt important (< 8%), some were frequently declared as having induced a reduction of meat consumption (> 88%). These motives were *“doctor’s advice”*, *“dislike for the taste of meat”* and *“healthier to avoid meat”* (of the 5% of participants who felt doctor’s advice important, 95.2% declared this motive as having induced a reduction of their meat consumption)*.* Other motives were less frequently felt important, and also less frequently declared as having induced a reduction of meat consumption, such as *“dislike of meat sight”* and “*budget concerns”*. For further details, see also Additional file 2.

#### Individual characteristics associated with change-inducing motives for meat reduction

**Fig. 4:**
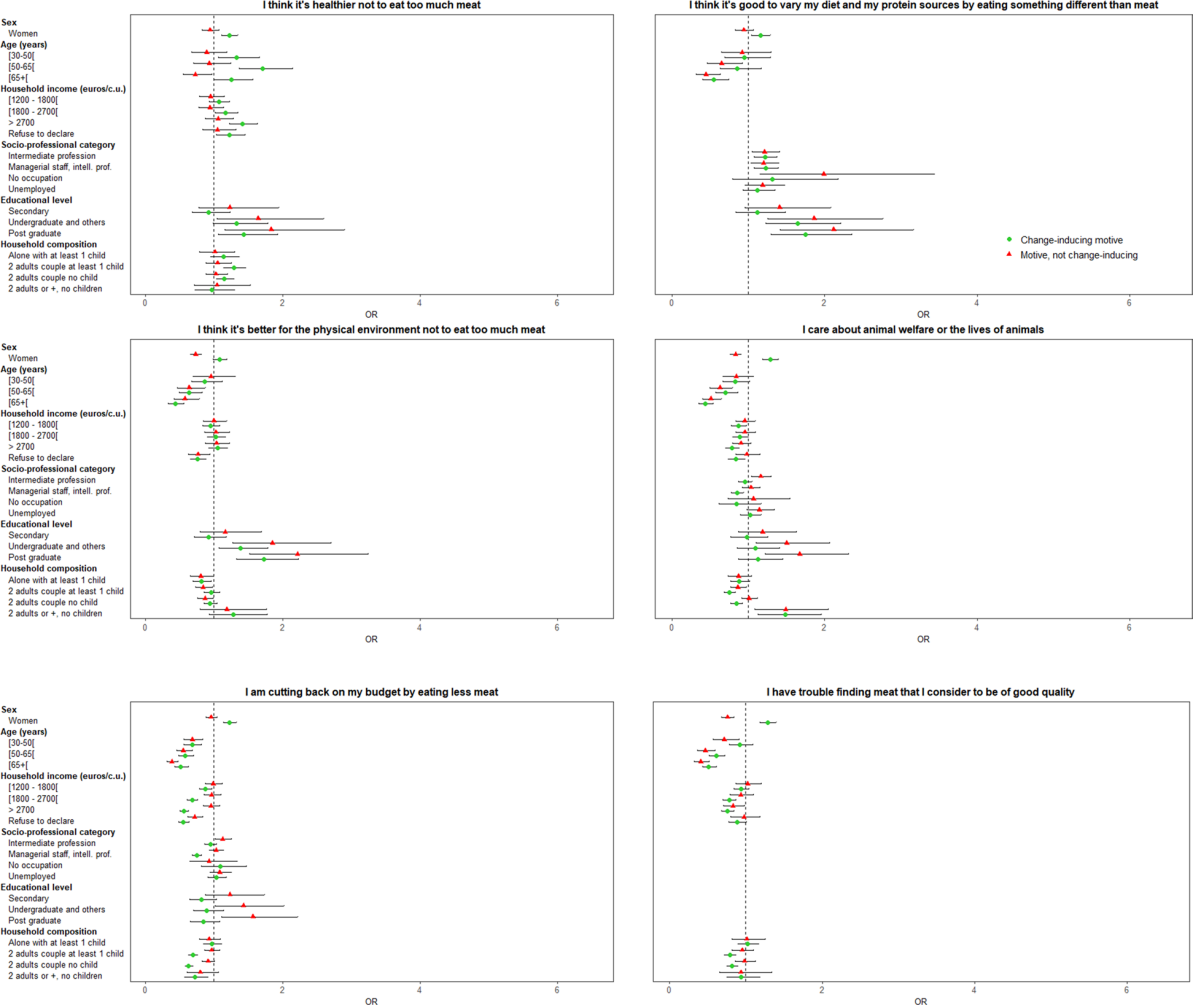

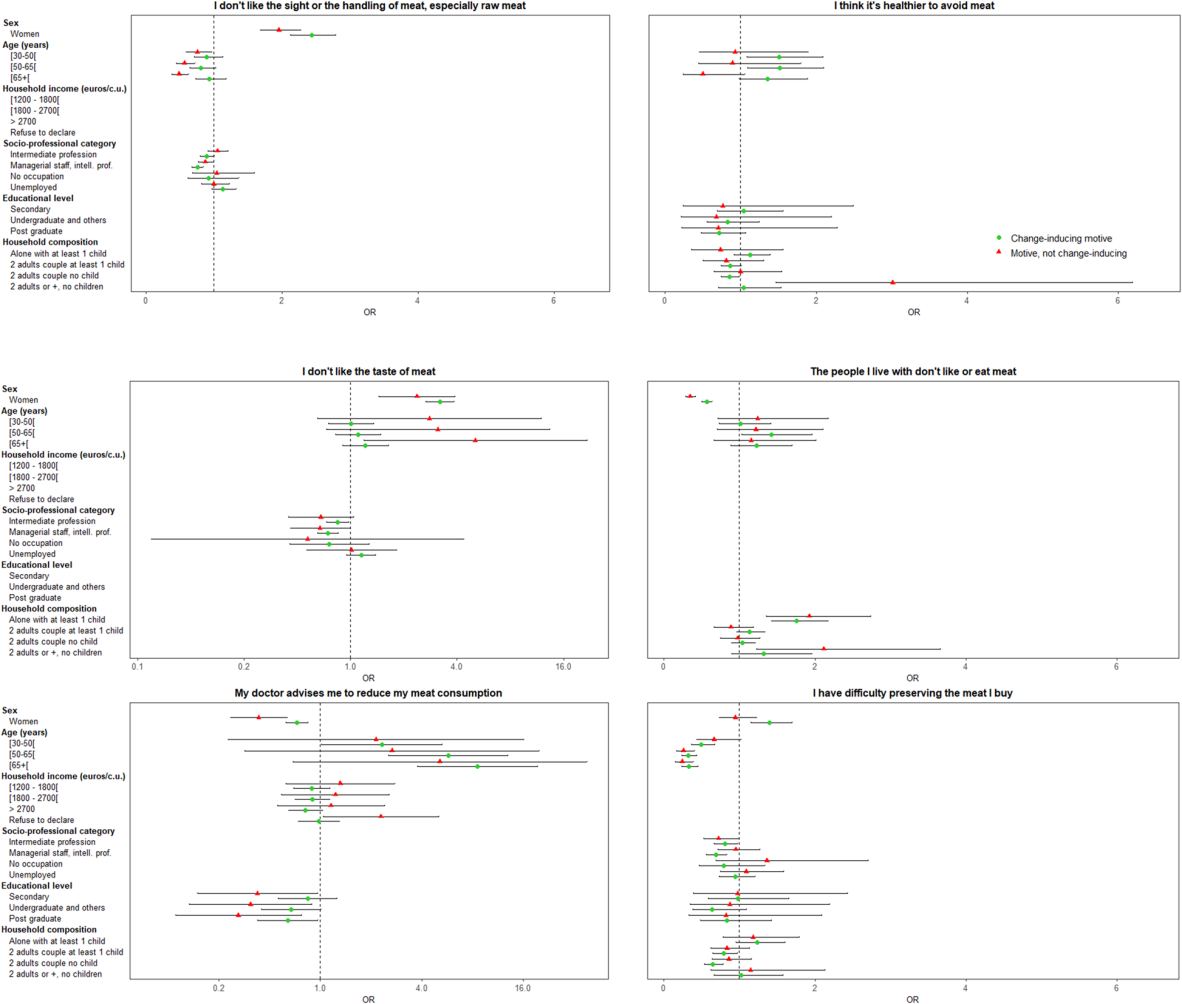
Association between individual characteristics and change-inducing motives for meat reduction (vs. “no motive”) / (motives ordered according to the frequency of individuals in the group “change-inducing motive”, multinomial logistic regression). For some figures, a logarithmic scale is used for easier reading of the results. All the models were also adjusted on BMI, size of the urban residence unit and declared latest weight-loss diet

Associated characteristics of individuals who declared a given motive as having induced a reduction of their meat consumption, compared to participants who did not declare the motive as important, are presented in Fig. 4. *“Dislike for the taste of meat”, “good to vary both diet and protein sources”, “healthier to limit meat”,* or *“animal welfare”* were more likely to be declared by women as having induced a reduction of their meat consumption, whereas *“doctor’s advice”* was more likely to be declared by men (all *p* <  0.001). “*Healthier to limit meat*” or “*to avoid meat*”, or “*doctor’s advice*” were more likely to be declared as having induced a reduction of meat consumption by older participants, whereas they were less likely to declare “*animal welfare*”, “*better for the physical environment to limit meat*”, and “*good to vary both diet and protein sources*”. (all *p* <  0.01). *“Healthier to limit meat”* was more likely to be declared as having induced a reduction of meat consumption by participants with a higher monthly income, but “*animal welfare”* was more likely to be declared by those with a lower monthly income (all *p* <  0.01). *“Good to vary both diet and protein sources”* was more likely to be declared as having induced a reduction of meat consumption by participants who had an intermediate or managerial occupation than occupations corresponding to self-employed, farmer, employee, manual worker, while they were less likely to declare *“dislike for the taste of meat”* (all *p* <  0.01). *“Animal welfare”* was also less likely to be declared by managerial occupations than other types of occupations (all *p* <  0.001). “*Good to vary both diet and protein sources”, “healthier”,* or “*better for the physical environment to limit meat”* were more likely to be declared as having induced a reduction of meat consumption by more highly educated individuals, whereas they were less likely to declare “*doctor’s advice”* (all *p* <  0.01). *“Better for the physical environment to limit meat”* was less likely to be declared as having induced a reduction of their meat consumption by participants living alone with at least one child (*p* <  0.05). *“Healthier to limit meat”* was more likely to be declared as having induced a reduction of their meat consumption by participants living as a couple with or without children, whereas “*animal welfare”* or *“healthier to avoid meat”* were less likely to be declared by these participants than by those living alone without children (all *p* <  0.01). Finally, “*animal welfare”* was more likely to be declared as having induced a reduction of their meat consumption by participants living with other adults but without children (*p* <  0.001)*.* Other motives such as “*budget”*, “*meat quality”* or *“meat preservation”* concerns*,* or “*dislike of meat sight”* were more likely to be declared by women, younger participants, those with lower socioeconomic status (at least for one characteristic between monthly incomes or socioprofessional category) and those living alone in the household (all *p* <  0.01).

### Legume increase

#### Change-inducing motives for a legume increase

**Fig. 5:**
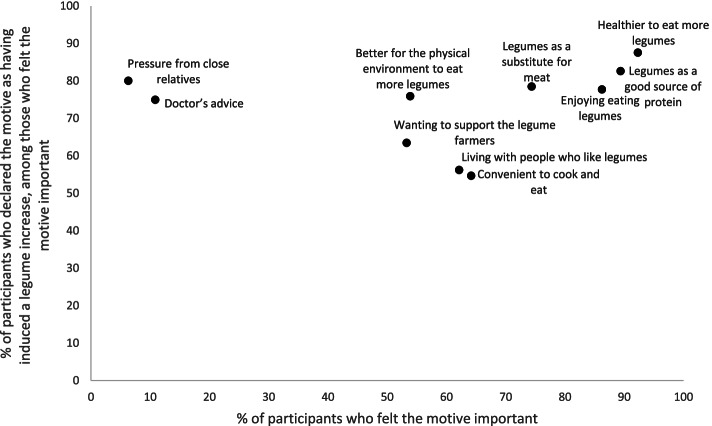
Comparison of the frequency of participants who declared the motive as having induced a legume increase, according to the frequency of participants who declared the motive as important. Legume increase sample. NutriNet-Santé study, 2018 (*n* = 16,446)

Among the motives frequently felt important (> 74%), four were frequently reported as having induced an increase of legume consumption (> 77%): *“healthier to eat more legumes”*, “*legumes as a good source of protein”*, *“enjoying eating legumes”*, and *“legumes as a substitute for meat”* (Fig. 5). Only 50% participants reported “*better for the physical environment to eat more legumes*” as an important motive for increasing legumes, but of these, 75.9% reported it as having induced an increase of their legume consumption. Two motives were less frequently felt important (< 11%), but were frequently declared as having induced an increase of legume consumption (> 74%), namely “*pressure from close relatives”* and *“doctor’s advice”*. For further details, see also Additional file 3.

#### Individual characteristics associated with change-inducing motives for a legume increase

**Fig. 6:**
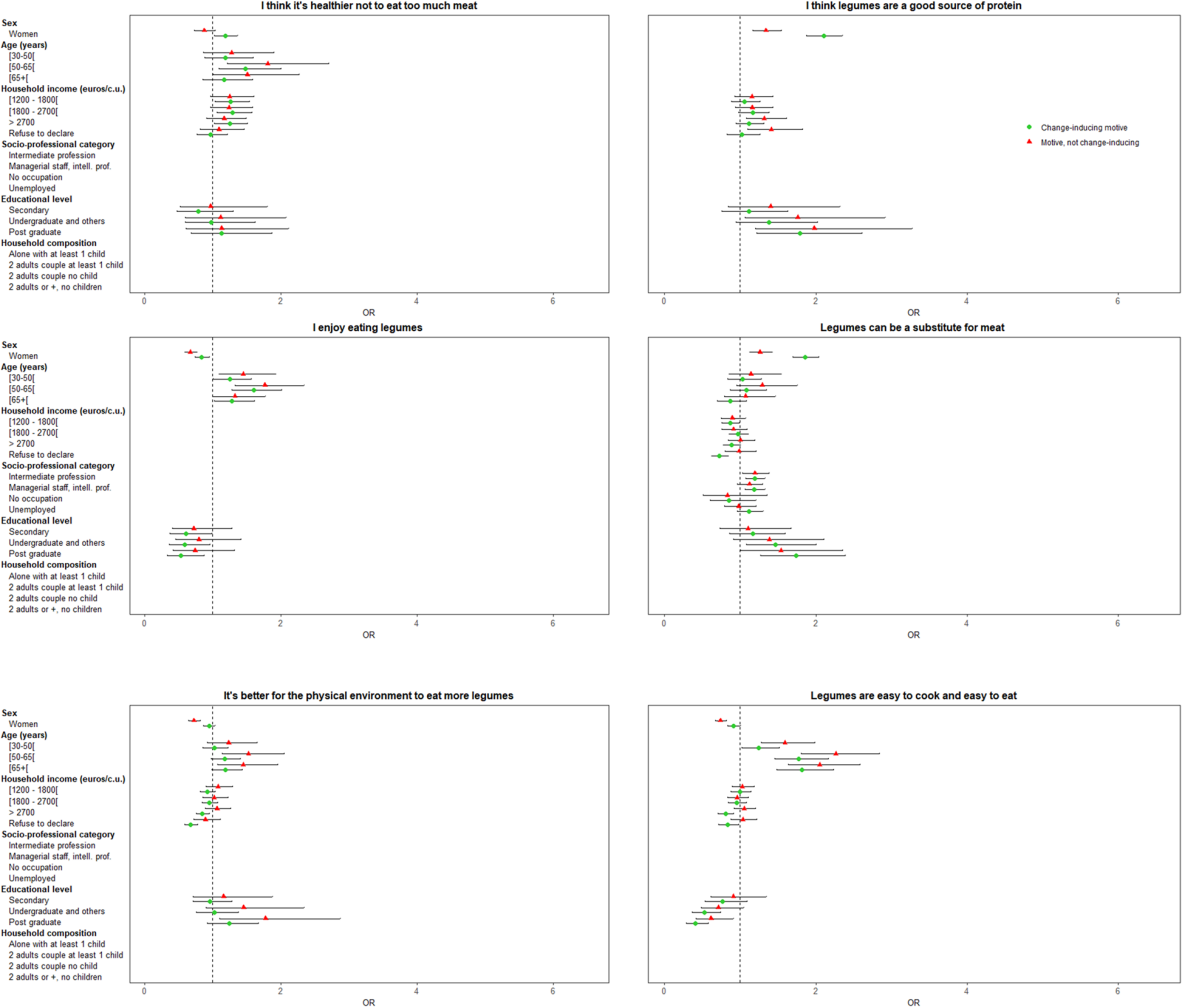

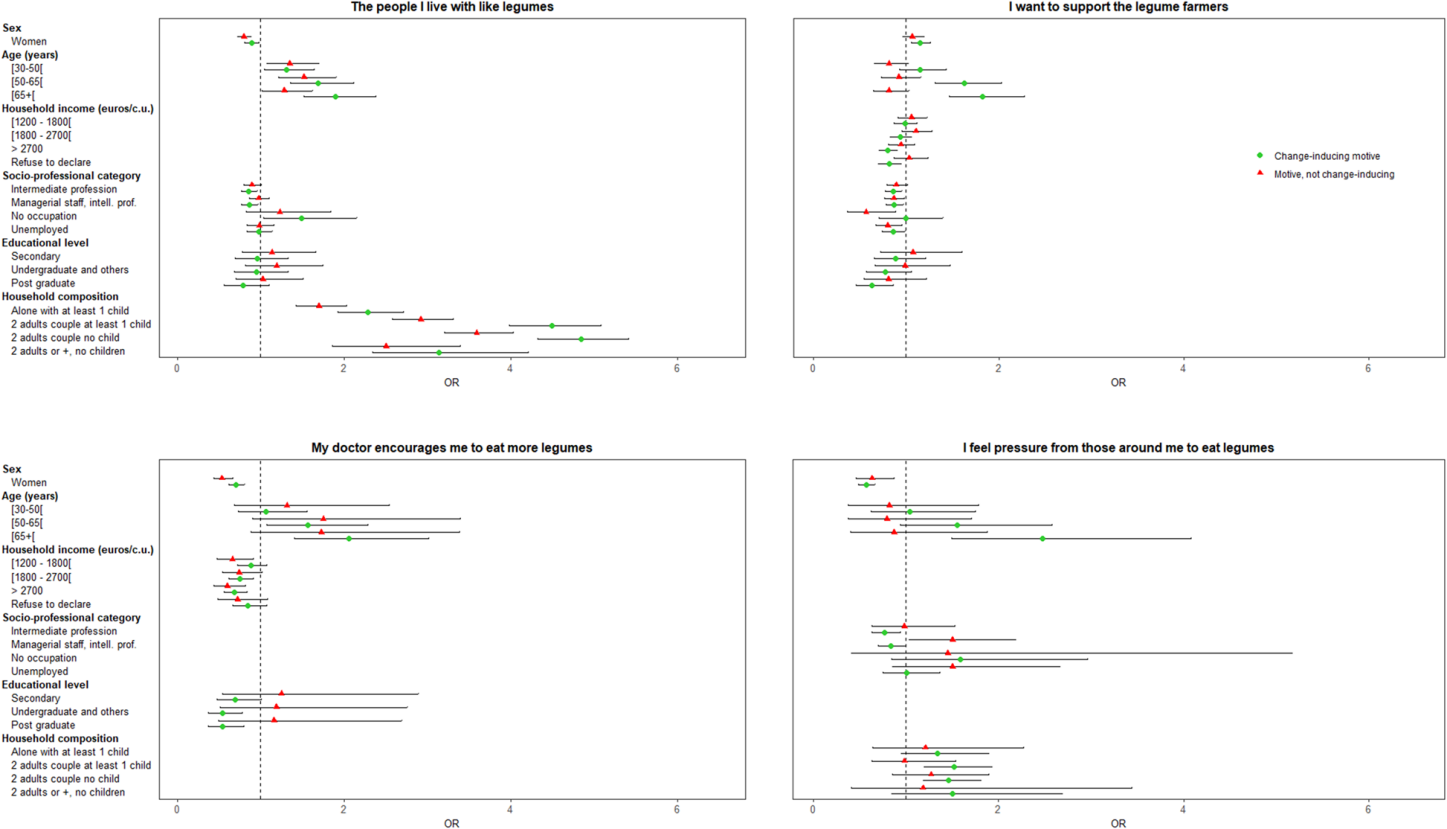
Association between individual characteristics and change-inducing motives for legume increase (vs. “no motive”) / (motives ordered according to the frequency of individuals in the group “change-inducing motive”, multinomial logistic regression)

Associated characteristics of individuals who declared a given motive as having induced an increase in their legume consumption, compared to those who did not declare that motive as important, are presented in Fig. 6.*“Healthier to eat more legumes”, “legumes as a substitute for meat”* or as *“a good source of protein”* were motives more likely to be declared by women as having induced an increase of their legume consumption, whereas “*enjoying eating legumes”; “pressure from close relatives”* or *“doctor’s advice”* were more likely to be declared by men (all *p* <  0.001).*“Enjoying eating legumes”, “healthier to eat more legumes”, “pressure from close relatives”* or *“doctor’s advice”* were more likely to be declared by older participants as having induced an increase of their legume consumption (all *p* <  0.001).*“Healthier to eat more legumes”* was more likely to be declared by participants with a higher monthly income as having induced an increase of their legume consumption, whereas *“better for the physical environment to eat more legumes”, “doctor’s advice”,* or *“legumes as a substitute for meat”* were more likely to be declared by those with a lower monthly income (all *p* <  0.05).

*“Legumes as a substitute for meat”* was more likely to be declared by intermediate professions or managerial staff as having induced an increase of their legume consumption, but *“pressure from close relatives”* was less likely to be declared by these participants (all *p* <  0.05)*. “Legumes as a substitute for meat”* or “*a good source of protein”* were more likely to be declared by highly educated participants as having induced an increase of their legume consumption, but “*enjoying eating legumes”* or “*doctor’s advice”* were less likely to be declared by these participants (all *p* <  0.01).*“Feeling pressure from close relatives”* was more likely to be declared as having induced an increase of legume consumption by participants living as a couple with or without children than by those living alone without children (*p* <  0.05). Finally, *“convenient to cook and eat legumes”* or “*living with people who like legumes”* were more likely to be declared by women, older participants, and those with lower socioeconomic position (at least, for one socioeconomic characteristic). “*Wanting to support legume farmers”* presented the same associations, except for sex (all *p* < 0.05).

### Rebalance in meat and legume consumptions

#### Individual characteristics associated with rebalance in meat and legume consumptions

Of the 22,567 participants who declared a meat reduction, 60.4% also declared an increase in their consumption of legumes (they rebalanced their meat and legume consumptions). Participants who declared a rebalance of their meat and legume consumptions were more likely to be women, younger participants, participants with higher incomes and those with higher educational levels (Table 2).

**Table 2:** Comparison of individual characteristics of participants who rebalanced their meat and legume consumptions and those who reduced their meat consumption but who did not increase their legume consumption (multivariable logistic regression, NutriNet-Santé 2009–2018, *n* = 22,567)

	Rebalance in meat and legumes (***n*** = 13,620)		Meat reduction and no legume increase (***n*** = 8947)			Rebalance in meat and legumes vs. Meat reduction and no legume increase		
	***n***	%	***n***	%	***p***	OR	95% CI	***p***
**Sex**					< 0.0001			< 0.0001
Men	2742	20.1	2396	26.8		REF	REF	
Women	10,878	79.9	6551	73.2		1.392	[1.3; 1.49]	
**Age**					< 0.0001			< 0.0001
[18–30[	530	3.9	295	3.3		REF	REF	
[30–50[	4286	31.5	2425	27.1		0.97	[0.83; 1.14]	
[50–65[	4921	36.1	3093	34.6		0.98	[0.83; 1.16]	
[65 + [	3883	28.5	3134	35.0		0.842	[0.71; 1]	
**Monthly household income classes**					0.0032			0.0084
< 1200 €	1790	13.1	1246	13.9		REF	REF	
1200–1800 €	2663	19.6	1782	19.9		1.08	[0.98; 1.19]	
1800–2700 €	3156	23.2	2095	23.4		1.091	[0.99; 1.2]	
> 2700 €	4736	34.8	2906	32.5		1.131	[1.03; 1.24]	
Refused to declare	1275	9.4	918	10.3		0.955	[0.85; 1.07]	
**Socioprofessional category**					< 0.0001			0.002
Self-employed, farmer, employee, manual worker	3178	23.3	2508	28.0		REF	REF	
Intermediate profession	3405	25.0	2073	23.2		1.163	[1.07; 1.26]	
Managerial staff, intellectual profession	5768	42.4	3572	39.9		1.031	[0.95; 1.12]	
No occupation	161	1.2	112	1.3		0.975	[0.75; 1.27]	
Unemployed	1108	8.1	682	7.6		1.094	[0.98; 1.22]	
**Educational level**					< 0.0001			< 0.0001
None or Primary	152	1.1	192	2.2		REF	REF	
Secondary	2884	21.2	2751	30.8		1.225	[0.98; 1.53]	
Undergraduate and others	4560	33.5	2733	30.6		1.788	[1.43; 2.23]	
Post graduate	6024	44.2	3271	36.6		2.009	[1.6; 2.52]	
**Household composition**					< 0.0001			
Alone without children	2450	18.0	1642	18.4		NA	NA	
Alone with at least one child	891	6.5	639	7.1		NA	NA	
Two adults living as a couple without children	5530	40.6	3821	42.7		NA	NA	
Two adults living as a couple with at least one child	4493	33.0	2681	30.0		NA	NA	
Two or more adults without children	256	1.9	164	1.8		NA	NA	
**Size of the urban residence unit**					0.0002			
Rural	2854	21.0	2002	22.4		NA	NA	
< 20,000 inhabitants	2035	14.9	1376	15.4		NA	NA	
20,000–200,000 inhabitants	2449	18.0	1707	19.1		NA	NA	
> 200,000 inhabitants	6282	46.1	3862	43.2		NA	NA	
**Latest weight-loss diet followed**					0.001			
No declared diet	5370	39.4	3317	37.1		NA	NA	
< 5 years	1005	7.4	651	7.3		NA	NA	
> 5 years	7245	53.2	4979	55.7		NA	NA	

^1^*p* for chi^2^ test

^2^*p* represented the overall significance of each variable (Type III analysis of effects), adjusted also on BMI (data not shown)

#### Change-inducing motives for the rebalance of meat and legume consumptions

Motives to rebalance consumption of meat and legumes are presented in Additional files 2 and 3. For participants rebalancing their meat and legume consumption, we observed similar frequencies of participants who declared a given motive as change-inducing as in the analysis of the meat reduction and the legume increase samples taken. Frequencies of those who declared a motive as change-inducing were even higher for participants rebalancing meat and legume consumption.

## Discussion

The present study aimed to describe the motives that could induce a rebalance of animal-based and plant-based products. Specifically, it focused on the changes in the consumption of meat and legumes, based on the declarations of a large sample of French adults. Consistent with our assumptions, we observed that some motives such as those related to health and nutrition, environment, and taste preferences, could be more effective in inducing a change in meat or legume consumptions than other motives. In addition, motives related to social influences, meat avoidance and meat dislike, though not frequently considered as important, were declared as change-inducing for the meat reduction or the legume increase. Sociodemographic and lifestyle characteristics were specifically associated with change-inducing motives for both meat and legume consumptions.

### Motives related to health and nutrition

In the present study, concerns for personal health and nutrition were the highest motives reported as having induced a change in meat and legume consumptions. Regarding meat, these results were consistent with findings from previous studies where “health” was a main motive reported by individuals who reduced their meat consumption [5, 7–9, 25–27]. A Finnish study suggested that the health motive could act as a “motivational force during the process of dietary change” [9]. Individuals with more health-oriented motives were more likely to pay attention to information from scientific sources [28]. In recent years, health messages promoting meat reduction based on international guidelines have been communicated to the public through the media and public health campaigns [29], to raise consumer awareness of these health issues. In comparison, consumers have had less health information on legumes. For instance, in France, it is only very recently (2017) that public health authorities have included a specific guideline on legumes in the official dietary guidelines [29]. Even so, we observed similar frequencies of participants who declared health and nutrition motives as change-inducing for the legume increase as for meat reduction.

In our study, participants frequently reported having increased their legume consumption because they replaced meat by legumes. However, a French study reported that outside of limited-budget or vegetarian diets, consumers mostly ate legumes in combination with meat, but not as a substitute for meat, as an additional protein source [30]. As the authors suggested, this may be related to culinary tradition in France, where some typical dishes with legumes also include meat or processed meat (e.g., “cassoulet”) [30]. It is thus important to connect dietary guidelines with practical information about how to eat and combine different plant-based foods in meals in order to foster new eating habits (e.g., French database on menus and recipes “La Fabrique à menus” [31]).

Of participants who declared a change in meat or legume consumption, women and highly educated individuals were more likely to report health and nutrition benefits as change-inducing motives. Overall, women are more interested in eating healthily, which includes chronic disease prevention and well-being, and are also more health conscious [32–34]. The association between higher educational level and better nutritional knowledge has already been documented [34–36]. Education may help to better understand and critically appropriate information. It may also raise concerns (e.g., how to maintain good health through dietary guidelines), influencing attitudes and behaviors [37], such as those regarding meat consumption. This might partly explain sociodemographic differences in animal and plant-based intakes previously observed in some NutriNet-Santé studies [16, 38].

Finally, older individuals were more likely to report health benefits as having induced a reduction in their meat consumption, while younger individuals were more likely to report motives related to nutrition. Older individuals were correspondingly more interested in healthy eating [33, 34, 39, 40], and were more likely to have healthier eating habits, including an increased consumption of plant-based foods [38].

### Motives related to the preservation of the physical environment

Preserving the physical environment by reducing meat consumption was frequently assessed as having induced a change in consumption. Previous studies have generally found that the environmental issue was not a frequently cited motive for reducing meat consumption [5, 7, 26, 41], but our results are consistent with a recent Canadian study in which about 60% of “meat reducers” reported environmental concerns as a reason for making conscious efforts to reduce meat consumption [5]. However, participants reporting a vegetarian diet were included in that study. The larger proportion in our study might be because the awareness of environmental pressure was more salient in 2018 when our questionnaire was sent. Over the past two decades, many scientists and politicians have warned of the current environmental crisis. Spurred by the media, public awareness of the impact of consumption choices on the environment is rather recent, notably through some political initiatives, such as the weekly meat- and fish-free meal in French school canteens [42]. Notably, the French High Council for Public Health mentioned for the first time in its latest report the need for greater awareness of the link between the nutritional and environmental aspects of dietary patterns [29]. On the other hand, our study population may be merely more aware of these issues. This possibility can be partly addressed by studying the associated sociodemographic and lifestyle characteristics.

First, in our study, younger participants were more likely to report the preservation of the environment as having induced a reduction in their meat consumption. However, a review including studies from 1987 to 2016 indicated that age was rarely associated with the environmental motive for reducing meat [43]. In recent years, younger individuals could have been more exposed to messages about climate change. In addition, a previous study based on the NutriNet-Santé cohort showed that future-oriented individuals were more likely to be younger [44]. As younger participants are more likely to be affected by environmental impacts on their future, they are probably more likely to be interested in and more motivated to change their behavior. This may therefore illustrate a “generational effect” rather than strictly a “younger age effect”.

As observed for health and nutrition, a higher educational level was associated with change-inducing motives related to physical environment for meat reduction. The education system provides general knowledge, and even specific skills related to environmental topics [45], and may help to better process environmental information. More highly educated participants may be thus more prone to acquire informal knowledge, either through the media such as by using the Internet and watching documentaries, or through social interaction [45]. In the literature, results from previous studies on the link between environmental motives for meat reduction and educational level are very heterogenous [46, 47]. This could be partly explained by the fact that environmental knowledge is defined and measured differently in studies.

For legumes, similarly to previous studies, preserving the environment did not appear as a strong motive, less than half the participants declaring this motive as important. A French focus group study showed that even with participants with good theoretical information such as the environmental impact of legumes, this did not seem to be reflected in their food choices, and in particular not by a higher consumption of legumes [48].

### Taste, pleasure and hedonic motives for rebalancing meat and legume consumption

The pleasure of eating legumes was frequently assessed as having induced an increase in legume consumption. This recalls previous studies where taste was described as a major reason for consuming legumes [49, 50]. This motive could also be a barrier to an increased consumption of legumes, as observed in relation to consumption in previous studies [30, 50].

In the present study, differences between certain sociodemographic characteristics and hedonic motives are highlighted for a declared increase in legume consumption. For instance, in our study, men were more likely to report the pleasure of eating legumes as having induced an increase in their consumption. Women were more likely to report legumes as a meat substitute and a good source of protein having induced an increase of their legume consumption. It would be of interest to investigate further which legumes-based meals men are more likely to prefer. Also, older participants were more likely to report the pleasure of eating legumes as having induced an increase in their legume consumption. In the literature, a higher consumption of legumes or plant-based foods was found in younger adults [49, 50]. However, it is well-known that taste preferences are closely related to social and cultural influences [51], and comparisons between consumption results from different countries seem to be of little relevance. In line with our results, there is a need to develop an interest in legumes among younger populations. Taste preferences are formed in childhood and continue throughout life, and younger individuals show greater plasticity in their preferences [52]. They are thus more favorable to learning and implementing new food behaviors. Like the very recent meat-free days in French school canteens, more initiatives focusing on legumes could be encouraged to develop interest and familiarity [42].

### Motives less frequently declared as important but having induced changes in consumption

In our study, although not frequently cited as important, certain motives such as those related to social influences, meat avoidance and meat dislike seem to be highly effective in inducing a change in consumption, even in smaller populations. For instance, from a public health and social marketing point of view, it is noteworthy that the “doctor’s advice” was a strong motive that induced a reduction in meat consumption and an increase in legume consumption. One hypothesis for why this motive was less frequently reported could be related to our study population. Healthy people do not consult general practitioners and therefore do not have the opportunity to receive such advice. Furthermore, only a few general practitioners provide nutritional advice and it is particularly targeted at patients considered at risk [53]. While they are ideally positioned for primary care, some barriers have been highlighted, such as lack of time, compensation, and confidence to provide nutritional care [53]. Another hypothesis can be advanced in the light of associated sociodemographic characteristics identified here. Men, older participants and participants with a lower educational level were more likely to report the doctor’s advice as having induced a reduction of their meat consumption. In line with our previous results, these participants may be less motivated by health and nutrition information, but may be more influenced by health professionals. General practitioners could thus efficiently contribute to the dissemination and the reinforcement of public health messages by advising a less aware or more resistant population. We note that two studies observed that individuals with a lower educational level were less likely to discuss health and nutrition information obtained on the Internet with health professionals [54, 55]. A similar association with older individuals was found only in the French study [54]. These individuals may therefore be more vulnerable to potentially misleading information on the Web, and thus engage in unbalanced eating behaviors. In addition to the greater role of health professionals and in order to address misinformation, public health institutions could strengthen the use of new online media such as social networks or mobile health applications. These could also be used for example to disseminate targeted nutrition messages on the benefits of rebalancing meat and legume consumptions.

### Strengths and contributions

Our findings bring new insights into the motives for changing food behaviors related to the consumption of meat and legumes. This is the first study to investigate change-inducing motives related to meat and legume consumptions and to make a detailed description of sociodemographic and lifestyle characteristics associated with these motives in France. We show that specific sociodemographic and lifestyle characteristics are differentially associated with motives. Considering motives less frequently declared could offer another way to induce a change in food consumption in specific subgroups, as some motives were declared more effective in inducing a change.

### Limits and future research

Participants from the NutriNet-Santé cohort are volunteers and so are probably more likely to be interested in nutrition topics. The external validity of this cross-sectional study may thus be affected as this population is not representative of the French population as a whole. Our population was certainly more aware of environmental topics owing to a large number of participants with high levels of education. The statistical power nevertheless enabled us to observe a wide range of different dietary behaviors. For instance, 7751 participants declared they were maintaining their meat consumption.

Regarding the evaluation of dietary changes, participants who were planning to change their consumption and those who had already done so were considered together in our study and a future study could examine the process of change more precisely. Moreover, the rebalance between meat and legumes was identified on the basis of the declarations of meat reduction and legume increase separately. A more direct approach could be considered in order to understand what people replace a food with (e.g.: asking individuals which plant-based food substituted animal-based food). Indeed, a recent study showed that many people seemed to be open to replacing meat with processed legumes [14], which may also have potential harmful health effects [56].

Our definition of meat included both ruminant meat and processed meat, and excluded other animal products such as poultry or fish. French and international dietary guidelines specify that to stay healthy and limit harmful effects on physical environment, adults need to limit their consumption of both red meat and processed meat. This is why we hypothesized that we could gather these two types of meat in our definition. Further studies describing in more detail the types of meat that individuals are cutting down on could complete our observations.

We found some associations between motives related to food environment and some individual characteristics such as the ease of finding good quality meat and younger participants. Thus, further studies could explore more broadly motives related to the wider food environment.

In this exploratory study, we chose to investigate the food choice motives in relation to changes in meat and legume consumptions, while other studies have explored factors influencing the reduction of meat consumption using theoretical constructs from models of behavior change. There could be similarities between the concept of food choice motives and the theoretical constructs of some behavior change models, as for “reflective motivation” in the COM-B model [57]. However, the conceptual framework of food choice motives and models of behavior change are different in terms of hypotheses and objectives, even if they all address factors that thought or found to have an impact on change [15]. This could therefore be a limitation in comparing our results with other studies. Further research could be conducted by combining the change-inducing motives with a theoretical model.

## Conclusions

In this cross-sectional study, we show that, concerns for personal health and for varying diet and protein sources by changing meat and legume consumptions were both important motives to induce a change in consumption, but the concern for environmental sustainability related to meat consumption, and the pleasure of eating legumes, were also important to change eating behaviors. All the motives were associated with specific sociodemographic and lifestyle characteristics, such as being a woman and being highly educated in the case of health motives. These differences may point to social inequalities in food choices, notably regarding health. Public campaigns on health and sustainability could develop new tools to reach other specific subgroups, for example by strengthening the role of primary care practitioners or by improving the use of recent online media (e.g., mobile health applications). Further work could explore various food behavior models applied to changes in meat consumption in longitudinal studies.

## Supplementary Information

**Additional file 1.** Motives for meat reduction and legume increase.

**Additional file 2.** Groups of motives for the reduction of meat consumption, in the samples of meat reduction and rebalance in meat and legumes, NutriNet-Santé study, 2018 (motives ordered according to the frequency of individuals in “Change-inducing motive” group).

**Additional file 3.** Groups of motives for the increase in legume consumption, in the samples of legume increase and rebalance in meat and legumes, NutriNet-Santé study, 2018 (motives ordered according to the frequency of individuals in “Change-inducing motive” group).

## Data Availability

The datasets generated and/or analyzed during the current study are not publicly available due protection under the protection of health data regulation set by the French National Commission for Information Technology and Liberties (Commission Nationale de l’Informatique et des Libertés, CNIL). The data are available upon reasonable request to the study’s operational manager, Nathalie Druesne-Pecollo (n.pecollo@eren.smbh.univ-paris13.fr), for review by the steering committee of the NutriNet-Santé study.

## Abbreviation

BMI: Body mass index
