# Supplementary material for: Rebalancing meat and legume consumption: change-inducing food choice motives and associated individual characteristics in non-vegetarian adults
Source: Int J Behav Nutr Phys Act. 2022 Sep 1;19:112. doi: 10.1186/s12966-022-01317-w (PMC9438278; doi:10.1186/s12966-022-01317-w)
Supplement: Supplementary file 1 — Additional file 1. Motives for meat reduction and legume increase. [file 12966_2022_1317_MOESM1_ESM.docx]

Additional file 1. Motives for meat reduction and legume increase

| **Meat reduction (*n* = 22,567)** | | |  |
| --- | --- | --- | --- |
| **Motive – full denomination** | **Motive – short denomination** | |  |
| I don't like the taste of meat | Dislike for the taste of meat | |  |
| I don't like the sight or the handling of meat, especially raw meat | Dislike of meat sight | |  |
| I think it's good to vary my diet and my protein sources by eating something different than meat | Good to vary both diet and protein sources | |  |
| I think it's healthier not to eat too much meat | Healthier to limit meat | |  |
| I think it's healthier to avoid meat | Healthier to avoid meat | |  |
| I care about animal welfare or the lives of animals | Animal welfare | |  |
| I think it's better for the physical environment not to eat too much meat | Better for the physical environment to limit meat | |  |
| The people I live with don't like or eat meat | Living with people who don’t eat meat | |  |
| My doctor advises me to reduce my meat consumption | Doctor’s advice | |  |
| I am cutting back on my budget by eating less meat | Budget concerns | |  |
| I have trouble finding meat that I consider to be of good quality: origin, traceability, hygiene, labeled meat, organic meat, or other quality criteria | Meat quality concerns | |  |
| I have difficulty preserving the meat I buy | Meat preserving concerns | |  |
| **Legume increase (*n* = 16,446)** | |  |  |
| **Motive– full denomination** | **Motive – short denomination** |  |  |
| I enjoy eating legumes | Enjoying eating legumes |  |  |
| It's better for the physical environment to eat more legumes | Better for the physical environment to eat more legumes |  |  |
| I think it's healthier to eat more legumes | Healthier to eat more legumes |  |  |
| I want to support the legume farmers | Wanting to support the legume farmers |  |  |
| The people I live with like legumes | Living with people who like legumes |  |  |
| I feel pressure from those around me to eat legumes | Pressure from close relatives |  |  |
| My doctor encourages me to eat more legumes | Doctor’s advice |  |  |
| Legumes are easy to cook and easy to eat | Convenient to cook and eat |  |  |
| Legumes can be a substitute for meat | Legumes as a substitute for meat |  |  |
| I think legumes are good source of protein | Legumes as a good source of protein |  |  |
