# Supplementary material for: Rebalancing meat and legume consumption: change-inducing food choice motives and associated individual characteristics in non-vegetarian adults
Source: Int J Behav Nutr Phys Act. 2022 Sep 1;19:112. doi: 10.1186/s12966-022-01317-w (PMC9438278; doi:10.1186/s12966-022-01317-w)
Supplement: Supplementary file 2 — Additional file 2. Groups of motives for the reduction of meat consumption, in the samples of meat reduction and rebalance in meat and legumes, NutriNet-Santé study, 2018 (motives ordered according to the frequency of individuals in “Change-inducing motive” group). [file 12966_2022_1317_MOESM2_ESM.docx]

Additional file 2. Groups of motives for the reduction of meat consumption, in the samples of meat reduction and rebalance in meat and legumes, NutriNet-Santé study, 2018 (motives ordered according to the frequency of individuals in “Change-inducing motive” group)

|  | **Meat reduction**  **(*n* = 22,567)** | |  | **Rebalance in meat and legumes**  **(*n* = 13,620)** | |  |  |
| --- | --- | --- | --- | --- | --- | --- | --- |
| **I think it's healthier not to eat too much meat** | | |  |  |  |  |  |
| *Total sample (n)* | *22567* |  |  | *13620* |  |  |  |
| No motive (%) |  | 12.3 |  |  | 8.8 |  |  |
| Motive, not change-inducing (%) |  | 10.5 |  |  | 8.2 |  |  |
| Change-inducing motive (%) |  | 77.2 |  |  | 83.0 |  |  |
|  |  |  |  |  |  |  |  |
| **I think it's good to vary my diet and my protein sources by eating something different than meat** | | | | | | | |
| *Total sample (n)* | *22567* |  |  | *13620* |  |  |  |
| No motive (%) |  | 10.3 |  |  | 6.8 |  |  |
| Motive, not change-inducing (%) |  | 17.1 |  |  | 13.2 |  |  |
| Change-inducing motive (%) |  | 72.6 |  |  | 80.0 |  |  |
|  |  |  |  |  |  |  |  |
| **I think it's better for the environment not to eat too much meat** | | | | | | |  |
| *Total sample (n)* | *22567* |  |  | *13620* |  |  |  |
| No motive (%) |  | 17.7 |  |  | 13.0 |  |  |
| Motive, not change-inducing (%) |  | 14.8 |  |  | 12.7 |  |  |
| Change-inducing motive (%) |  | 67.5 |  |  | 74.4 |  |  |
|  |  |  |  |  |  |  |  |
| **I care about animal welfare or the lives of animals** | | | |  |  |  |  |
| *Total sample (n)* | *22567* |  |  | *13620* |  |  |  |
| No motive (%) |  | 30.9 |  |  | 27.3 |  |  |
| Motive, not change-inducing (%) |  | 23.8 |  |  | 22.5 |  |  |
| Change-inducing motive (%) |  | 45.4 |  |  | 50.2 |  |  |
|  |  |  |  |  |  |  |  |
| **I am cutting back on my budget by eating less meat** | | | |  |  |  |  |
| *Total sample (n)* | *22567* |  |  | *13620* |  |  |  |
| No motive (%) |  | 48.6 |  |  | 45.5 |  |  |
| Motive, not change-inducing (%) |  | 21.7 |  |  | 22.4 |  |  |
| Change-inducing motive (%) |  | 29.7 |  |  | 32.2 |  |  |
|  |  |  |  |  |  |  |  |
| **I have trouble finding meat that I consider to be of good quality: origin, traceability, hygiene, labeled meat, organic meat, or other quality criteria** | | | | | | | |
| *Total sample (n)* | *22567* |  |  | *13620* |  |  |  |
| No motive (%) |  | 66.3 |  |  | 65.2 |  |  |
| Motive, not change-inducing (%) |  | 9.0 |  |  | 8.1 |  |  |
| Change-inducing motive (%) |  | 24.7 |  |  | 26.7 |  |  |
|  |  |  |  |  |  |  |  |
| **I don't like the sight or the handling of meat, especially raw meat** | | | | | | |  |
| *Total sample (n)* | *22567* |  |  | *13620* |  |  |  |
| No motive (%) |  | 82.3 |  |  | 81.3 |  |  |
| Motive, not change-inducing (%) |  | 7.3 |  |  | 7.5 |  |  |
| Change-inducing motive (%) |  | 10.5 |  |  | 11.2 |  |  |
|  |  |  |  |  |  |  |  |
| **I think it's healthier to avoid meat** |  |  |  |  |  |  |  |
| *Total sample (n)* | *22567* |  |  | *13620* |  |  |  |
| No motive (%) |  | 92.2 |  |  | 91.2 |  |  |
| Motive, not change-inducing (%) |  | 0.7 |  |  | 0.7 |  |  |
| Change-inducing motive (%) |  | 7.1 |  |  | 8.1 |  |  |
|  |  |  |  |  |  |  |  |
| **I don't like the taste of meat** |  |  |  |  |  |  |  |
| *Total sample (n)* | *22567* |  |  | *13620* |  |  |  |
| No motive (%) |  | 92.4 |  |  | 81.3 |  |  |
| Motive, not change-inducing (%) |  | 0.7 |  |  | 0.7 |  |  |
| Change-inducing motive (%) |  | 6.9 |  |  | 7.1 |  |  |
|  |  |  |  |  |  |  |  |
| **The people I live with don't like or eat meat** | | |  |  |  |  |  |
| *Total sample (n)* | *22567* |  |  | *13620* |  |  |  |
| No motive (%) |  | 90.9 |  |  | 90.2 |  |  |
| Motive, not change-inducing (%) |  | 2.2 |  |  | 2.0 |  |  |
| Change-inducing motive (%) |  | 6.9 |  |  | 7.8 |  |  |
|  |  |  |  |  |  |  |  |
| **My doctor advises me to reduce my meat consumption** | | | | |  |  |  |
| *Total sample (n)* | *22567* |  |  | *13620* |  |  |  |
| No motive (%) |  | 95.2 |  |  | 94.9 |  |  |
| Motive, not change-inducing (%) |  | 0.5 |  |  | 0.5 |  |  |
| Change-inducing motive (%) |  | 4.3 |  |  | 4.7 |  |  |
|  |  |  |  |  |  |  |  |
| **I have difficulty preserving the meat I buy** | | |  |  |  |  |  |
| *Total sample (n)* | *22567* |  |  | *13620* |  |  |  |
| No motive (%) |  | 94.4 |  |  | 94.8 |  |  |
| Motive, not change-inducing (%) |  | 1.7 |  |  | 1.6 |  |  |
| Change-inducing motive (%) |  | 3.8 |  |  | 3.7 |  |  |
