# Supplementary material for: Rebalancing meat and legume consumption: change-inducing food choice motives and associated individual characteristics in non-vegetarian adults
Source: Int J Behav Nutr Phys Act. 2022 Sep 1;19:112. doi: 10.1186/s12966-022-01317-w (PMC9438278; doi:10.1186/s12966-022-01317-w)
Supplement: Supplementary file 3 — Additional file 3. Groups of motives for the increase in legume consumption, in the samples of legume increase and rebalance in meat and legumes, NutriNet-Santé study, 2018 (motives ordered according to the frequency of individuals in “Change-inducing motive” group). [file 12966_2022_1317_MOESM3_ESM.docx]

Additional file 3. Groups of motives for the increase in legume consumption, in the samples of legume increase and rebalance in meat and legumes, NutriNet-Santé study, 2018 (motives ordered according to the frequency of individuals in “Change-inducing motive” group)

|  | **Legume increase**  **(*n* = 16,446)** | |  | **Rebalance in meat and legumes**  **(*n* = 13,620)** | |  |
| --- | --- | --- | --- | --- | --- | --- |
| **I think it's healthier to eat more legumes** | | |  |  |  |  |
| *Total sample (n)* | *16446* |  |  | *13620* |  |  |
| No motive (%) |  | 7.7 |  |  | 6.9 |  |
| Motive, not change-inducing (%) |  | 11.5 |  |  | 10.5 |  |
| Change-inducing motive (%) |  | 80.8 |  |  | 82.7 |  |
|  |  |  |  |  |  |  |
| **I think legumes are a good source of protein** | | |  |  |  |  |
| *Total sample (n)* | *16446* |  |  | *13620* |  |  |
| No motive (%) |  | 10.6 |  |  | 9.0 |  |
| Motive, not change-inducing (%) |  | 15.6 |  |  | 13.6 |  |
| Change-inducing motive (%) |  | 73.8 |  |  | 77.5 |  |
|  |  |  |  |  |  |  |
| **I enjoy eating legumes** |  |  |  |  |  |  |
| *Total sample (n)* | *16446* |  |  | *13620* |  |  |
| No motive (%) |  | 13.8 |  |  | 13.3 |  |
| Motive, not change-inducing (%) |  | 19.2 |  |  | 18.7 |  |
| Change-inducing motive (%) |  | 67.0 |  |  | 68.1 |  |
|  |  |  |  |  |  |  |
| **Legumes can be a substitute for meat** | | |  |  |  |  |
| *Total sample (n)* | *16446* |  |  | *13620* |  |  |
| No motive (%) |  | 25.7 |  |  | 22.4 |  |
| Motive, not change-inducing (%) |  | 16.0 |  |  | 14.5 |  |
| Change-inducing motive (%) |  | 58.3 |  |  | 63.1 |  |
|  |  |  |  |  |  |  |
| **It's better for the environment to eat more legumes** | | |  |  |  |  |
| *Total sample (n)* | *16446* |  |  | *13620* |  |  |
| No motive (%) |  | 46.1 |  |  | 43.3 |  |
| Motive, not change-inducing (%) |  | 13.0 |  |  | 12.8 |  |
| Change-inducing motive (%) |  | 40.9 |  |  | 43.9 |  |
|  |  |  |  |  |  |  |
| **Legumes are easy to cook and easy to eat** | | |  |  |  |  |
| *Total sample (n)* | *16446* |  |  | *13620* |  |  |
| No motive (%) |  | 35.8 |  |  | 35.5 |  |
| Motive, not change-inducing (%) |  | 29.1 |  |  | 28.4 |  |
| Change-inducing motive (%) |  | 35.1 |  |  | 36.1 |  |
|  |  |  |  |  |  |  |
| **The people I live with like legumes** | | |  |  |  |  |
| *Total sample (n)* | *16446* |  |  | *13620* |  |  |
| No motive (%) |  | 37.9 |  |  | 36.6 |  |
| Motive, not change-inducing (%) |  | 27.2 |  |  | 27.2 |  |
| Change-inducing motive (%) |  | 34.9 |  |  | 36.2 |  |
|  |  |  |  |  |  |  |
| **I want to support the legume farmers** | | |  |  |  |  |
| *Total sample (n)* | *16446* |  |  | *13620* |  |  |
| No motive (%) |  | 46.7 |  |  | 44.7 |  |
| Motive, not change-inducing (%) |  | 19.5 |  |  | 19.6 |  |
| Change-inducing motive (%) |  | 33.8 |  |  | 35.7 |  |
|  |  |  |  |  |  |  |
| **My doctor encourages me to eat more legumes** | | |  |  |  |  |
| *Total sample (n)* | *16446* |  |  | *13620* |  |  |
| No motive (%) |  | 89.2 |  |  | 90.0 |  |
| Motive, not change-inducing (%) |  | 2.7 |  |  | 2.4 |  |
| Change-inducing motive (%) |  | 8.1 |  |  | 7.7 |  |
|  |  |  |  |  |  |  |
| **I feel pressure from those around me to eat legumes** | | | |  |  |  |
| *Total sample (n)* | *16446* |  |  | *13620* |  |  |
| No motive (%) |  | 93.7 |  |  | 93.9 |  |
| Motive, not change-inducing (%) |  | 1.3 |  |  | 1.2 |  |
| Change-inducing motive (%) |  | 5.0 |  |  | 4.9 |  |
